# Modular Inducible Multigene Expression System for Filamentous Fungi

**DOI:** 10.1128/spectrum.03670-22

**Published:** 2022-11-09

**Authors:** Clara Baldin, Alexander Kühbacher, Petra Merschak, Johannes Wagener, Fabio Gsaller

**Affiliations:** a Institute of Molecular Biology, Biocenter Innsbruck, Medical University of Innsbruck, Innsbruck, Austria; b Department of Clinical Microbiology, School of Medicine, Trinity College Dublin, The University of Dublin, Dublin, Ireland; Universidade de Sao Paulo

**Keywords:** antifungal resistance, *Aspergillus fumigatus*, ergosterol biosynthesis, filamentous fungi, inducible promoters, multigene expression

## Abstract

Inducible promoters are indispensable elements when considering the possibility to modulate gene expression on demand. Desirable traits of conditional expression systems include their capacity for tight downregulation, high overexpression, and in some instances for fine-tuning, to achieve a desired product’s stoichiometry. Although the number of inducible systems is slowly increasing, suitable promoters comprising these features are rare. To date, the concomitant use of multiple regulatable promoter platforms for controlled multigene expression has been poorly explored. This work provides pioneer work in the human pathogenic fungus Aspergillus fumigatus, wherein we investigated different inducible systems, elucidated three candidate promoters, and proved for the first time that up to three systems can be used simultaneously without interfering with each other. Proof of concept was obtained by conditionally expressing three antifungal drug targets within the ergosterol biosynthetic pathway under the control of the xylose-inducible *PxylP* system, the tetracycline-dependent Tet-On system, and the thiamine-repressible *PthiA* system.

**IMPORTANCE** In recent years, inducible promoters have gained increasing interest for industrial or laboratory use and have become key instruments for protein expression, synthetic biology, and metabolic engineering. Constitutive, high-expressing promoters can be used to achieve high expression yields; however, the continuous overexpression of specific proteins can lead to an unpredictable metabolic burden. To prevent undesirable effects on the expression host’s metabolism, the utilization of tunable systems that allow expression of a gene product on demand is indispensable. Here, we elucidated several excellent tunable promoter systems and verified that each can be independently induced in a single strain to ultimately develop a unique conditional multigene expression system. This highly efficient, modular toolbox has the potential to significantly advance applications in fundamental as well as applied research in which regulatable expression of several genes is a key requirement.

## INTRODUCTION

Filamentous fungi are gaining increasing visibility in a variety of fields. In the industrial sector, they are considered economically relevant producers of different types of primary and secondary metabolites, enzymes, and small bioactive compounds, while in the medical field the infection rate and bad outcomes due to fungal pathogens are on the rise (1–5). A relevant part of optimizing biotechnological strains, as well as in characterizing clinically relevant strains, relies on the extensive use of tools for genetic engineering. Initially, biotechnological production relied mainly on bacterial and yeast expression, but the quick development of synthetic biology in various species allowed closing the technological gap that separated filamentous fungi from simpler expression systems (2, 6, 7). At the present time, filamentous fungi are considered an excellent tool for protein production, due to their posttranslational modification system, their natural capacity to secrete the desired product, and their ability to express entire biosynthetic gene clusters (8, 9). Moreover, the use of fungal species as biocontrol agents to counteract phytopathogens and plant parasites offers a valuable alternative to reduce the usage of chemically synthesized pesticides (10). The continuous implementation of genetic toolboxes has also allowed more sophisticated approaches to study human fungal pathogens. The possibility to specifically mutate a single gene at a time, without relying on random mutagenesis strategies followed by deep screening, improved significantly the characterization of genes involved in virulence and the consequent identification of putative drug targets (11, 12).

In addition to genetic manipulation tools, it has already been some years since the great potential of influencing gene regulation became explicit. The DNA sequence immediately upstream of a start codon is the one responsible for controlling the expression of the adjacent gene and is defined as the promoter region. If at first strong constitutive promoters were favored for both protein production and expressing resistance markers in laboratory studies, the possibility of adjusting the expression of the desired target acquired increased popularity more recently (13–15). Several inducible promoters have been identified and successfully used in different species of filamentous fungi; however, the desirable characteristics of strong upregulation and tight regulation, cost-efficient induction, and fine-tuned expression are not often satisfied (16). Depending on the aim and on the required culture conditions, the promoter’s inducing agent has to be carefully evaluated. Some compounds, or the overexpressed product itself, might exert toxic effects in specific organisms (17, 18). In other cases, the cost of the inducing substance could determine the fate of a promoter as not economically suitable for industrial production (19, 20). To study genes that are expressed at low levels or essential genes, instead, the leakiness of the promoter, often resulting in minimal constitutive activity, can prevent the efficient characterization of the target (19).

The genus Aspergillus comprises several filamentous fungal species, some of which are commonly used in industrial production, e.g., Aspergillus nidulans (21), Aspergillus oryzae (22), and Aspergillus niger (23), while others are classified and studied as human pathogens, like Aspergillus fumigatus (24) or Aspergillus flavus (25). Various inducible promoters have been identified and tested in Aspergillus spp. (for a detailed review, see Kück et al. [16]), each presenting its own advantages and disadvantages and resulting in a reduced pool of candidates that are considered efficient systems. Our laboratory mostly works with A. fumigatus, which represents one of the most relevant airborne fungal pathogens and allergens in the world (3, 26). This study presents a preliminary analysis comparing different inducible promoters that are available for A. fumigatus, in order to better characterize them and to identify the most suitable candidates. Inducible promoters commonly used in this species are currently represented by the xylanase promoter *PxylP* from Penicillium chrysogenum (27) and distinct Tet-On systems (20, 28). To expand the pool of inducible systems in our study, we decided to include another inducible promoter, *PthiA* from A. oryzae (29). This promoter has been successfully tested in different Aspergillus species for conditional gene expression (30, 31), and we decided to further explore its potential. Using green fluorescent protein with an S-to-T change at position 65 (GFP^S65T^) as a reporter (32), we analyzed the activity profile of four promoter systems, *PxylP*, two versions of Tet-On, and *PthiA*, and we provide evidence that three of our candidates fulfill the requirement for high and low expression as well as fine-tunability. Importantly, our results demonstrate that the induction or repression of each system does not interfere with the others. As a key application, we further tested their suitability for the conditional expression of multiple genes within a single strain. For this purpose, we selected three genes encoding enzymes of the ergosterol biosynthetic pathway (33) with known selective inhibitors, azoles, statins, and allylamines, targeting Cyp51, Hmg1, and Erg1, respectively (34–36). For site-directed integration of the expression cassettes containing each gene under the control of a different inducible system, we exploited the previously discovered counterselectable marker loci *fcyB*, *fcyA*, and *uprt* (37). Through resistance analysis, we were able to unveil for the first time a modular toolbox in this species that allows the simultaneous but still independent conditional expression of three different genes.

Due to the similarities among different filamentous fungi, we foresee that our study might serve as a pilot to implement this modular system in other organisms, including further medically and biotechnologically relevant species.

## RESULTS

### Discrete expression profiling of inducible promoters in A. fumigatus.

According to the literature, two of the most commonly used inducible systems in A. fumigatus are represented by *PxylP* and Tet-On (20, 27, 28). *PthiA*, on the contrary, has not been widely used in general and the regulation of this promoter is still unclear. Enticed by the *PthiA* promoter’s strength and inducibility, which can be deduced from other studies (30, 38), we decided to include it in our work to further evaluate its suitability as molecular tool. Adequate comparison of the regulation profile of these promoters required the exclusion of any possible locus-related effects; hence, we selected the previously described endogenous marker *fcyB* (37) for the integration of the constructs. As a reporter to verify the expression profile of the studied promoters, we selected GFP^S65T^ (32), abbreviated here for simplicity as sGFP. As a reference for the expression, we included the well-characterized and strong constitutive promoter *PgpdA* from Aspergillus nidulans (39, 40), while to set a baseline and evaluate unspecific GFP fluorescence, we considered the background signal generated by the wild-type (wt) strain.

**FIG 1:**
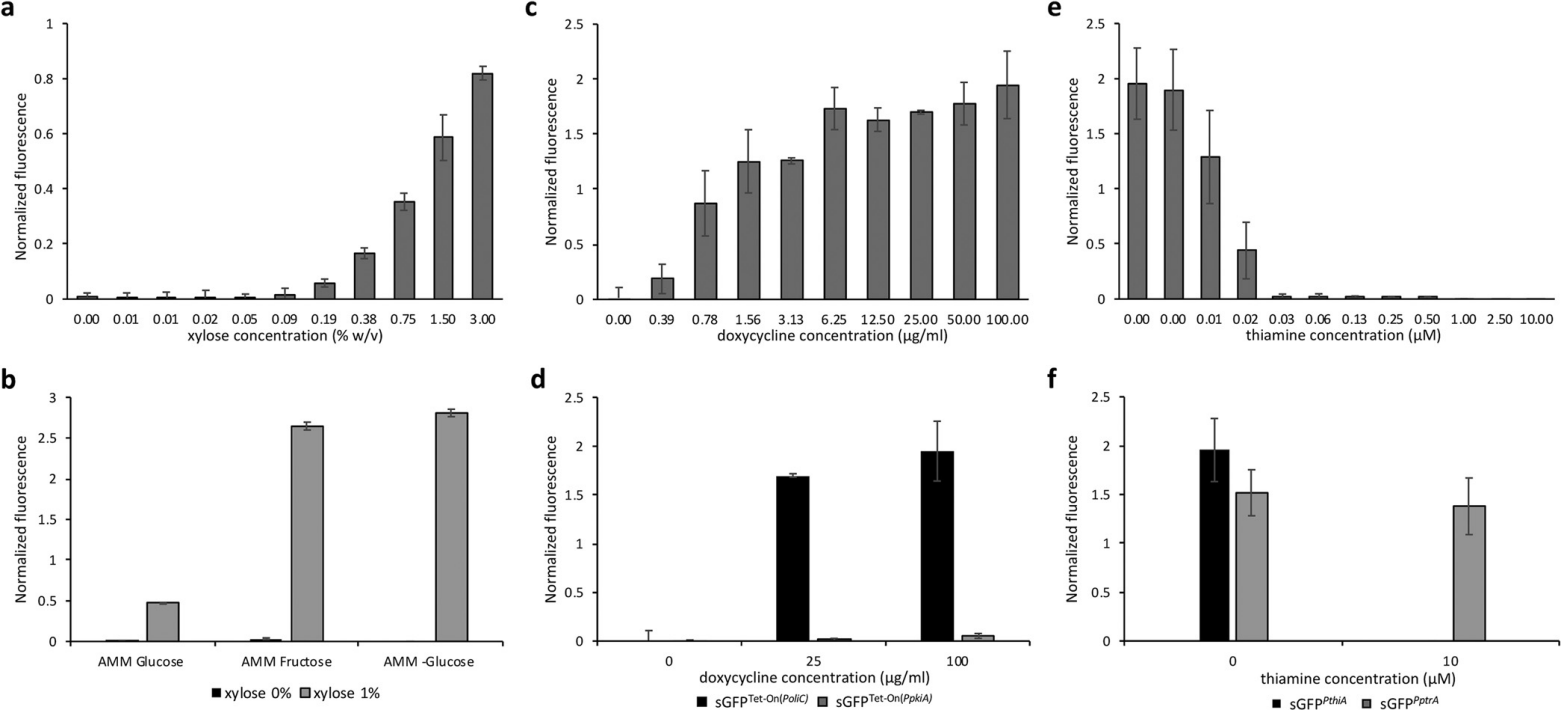
Fluorescence-based evaluation of inducible promoters’ expression profiles in comparison to *PgpdA*. (a, c, and e) Expression generated by *PxylP* (a), Tet-On(*PoliC*) (c), and *PthiA* (e) in a titration analysis conducted with xylose, doxycycline, and thiamine, respectively, in *Aspergillus* minimal medium (AMM). (b) Effects of different carbon sources on *PxylP* induction. (d) Induction variability between Tet-On(*PoliC*) and Tet-On(*PpkiA*) with 25 μg/mL and 100 μg/mL doxycycline. (f) Thiamine-repressing effect on *PthiA* in comparison to *PptrA.* sGFP fluorescence was normalized to that generated by *PgpdA*. To set a baseline and evaluate unspecific GFP fluorescence, the background signal generated by wt was subtracted.

Initially, we analyzed each promoter separately, to better establish the optimal conditions for their induction or repression. *PxylP* has been widely used in A. fumigatus (38, 41–44), and the general conditions to obtain a high level of expression were set to 1% glucose and 1% xylose in minimal medium, while glucose alone would be suitable to efficiently downregulate *PxylP*-driven gene expression. We conducted a titration analysis using 1% glucose and xylose in a concentration range from 0% to 3%, and we confirmed, as previously described (38), that a concentration as low as 0.09% xylose was sufficient for induction (Fig. 1a). Induction of *PxylP* with 1% xylose reached about 0.5-fold that induced by *PgpdA*. Increasing the xylose concentration to 3% elevated the expression level to about 0.8-fold that achieved with *PgpdA.*

As glucose is expected to induce carbon catabolite repression and, therefore, diminish *PxylP* activity (27), we further monitored its activity in the presence of 1% xylose without an additional carbon source, as well as in 1% xylose plus 1% fructose. Both conditions led to a severe increase in *PxylP* activity (>2.5-fold that of *PgpdA*); however, without glucose, residual expression of the reporter could also be detected under repressing conditions (Fig. 1b).

To date, various versions of the Tet-On system have been successfully used in A. fumigatus (19, 45, 46). We decided to compare two previously constructed Tet-On cassettes: Tet-On(*PoliC*) (45) and Tet-On(*PpkiA*) (46). Both cassettes have the same origin, pJW123 (45), and harbor a promoter that drives expression of the reverse tetracycline transactivator (rtTA), which is followed by a terminator region and a chimeric rtTA-dependent *tetO*-Pmin promoter. The main differences are the following: (i) the promoter that drives expression of the rtTA, which is A. nidulans *PgpdA* in the Tet-On(*PoliC*) cassette and A. niger *PpkiA* in the Tet-On(*PpkiA*) cassette; (ii) the chimeric *tetO*-Pmin promoter, which contains a part of A. nidulans *PgpdA* in the case of the Tet-On(*PpkiA*) cassette or a part of A. nidulans *PoliC* in the case of the Tet-On(*PoliC*) cassette. We performed a titration assay for both variants using doxycycline in a concentration range of 0 to 100 μg/mL. For the Tet-On(*PoliC*) system, 6 μg/mL seemed already sufficient to achieve its highest activity, which was >1.7-fold higher than that of *PgpdA* (Fig. 1c). Instead, for the Tet-On(*PpkiA*) system, even 100 μg/mL inducer was not sufficient to promote high expression (Fig. 1d).

To monitor the tunability of *PthiA*, a titration experiment was carried out using thiamine concentrations in a range between 0 and 10 μM. In contrast to the two other systems, *PthiA* is active in standard Aspergillus minimal medium (AMM), while thiamine acts as a repressor via a riboswitch-based mechanism (47). In the absence of thiamine, the activity of *PthiA* was increased almost 2-fold compared to that for *PgpdA*. A small concentration of thiamine, equal to 0.03 μM, was sufficient to almost completely silence the promoter (Fig. 1e). We further compared the native *PthiA* promoter with the point-mutated variant *PptrA*, which is part of the *ptrA* marker cassette (29), and verified that the mutation in the 5′-untranslated region (UTR) led to a lack of thiamine regulation. *PptrA* showed an expression level similar to *PthiA* in the absence of thiamine, but as expected, the presence of the repressor thiamine did not affect *PptrA* induction (Fig. 1f).

### Transcript level-based assessment of the promoters’ inducibility and leakiness.

In the next step, we monitored *PxylP*-mediated gene expression under standard conditions, i.e., in the presence of 1% glucose to minimize the promoter’s leakiness (Fig. 1b) and with 1% glucose plus 1% xylose for induction. Regarding the Tet-On systems, due to the very low expression obtained with Tet-On(*PpkiA*) during inducing conditions in comparison with the other promoter, this system appeared unsuitable for approaches where high overexpression was required. Hence, we selected Tet-On(*PoliC*) for further investigations. The concentration of doxycycline for induction was set at 10 μg/mL. Under the assumption that an adequate concentration of thiamine can eventually repress *PthiA* for the required period of time, and considering that especially for overexpression the results were quite promising, we included this promoter in the subsequent experiments and chose a concentration of 10 μM thiamine for repressing conditions.

**FIG 2:**
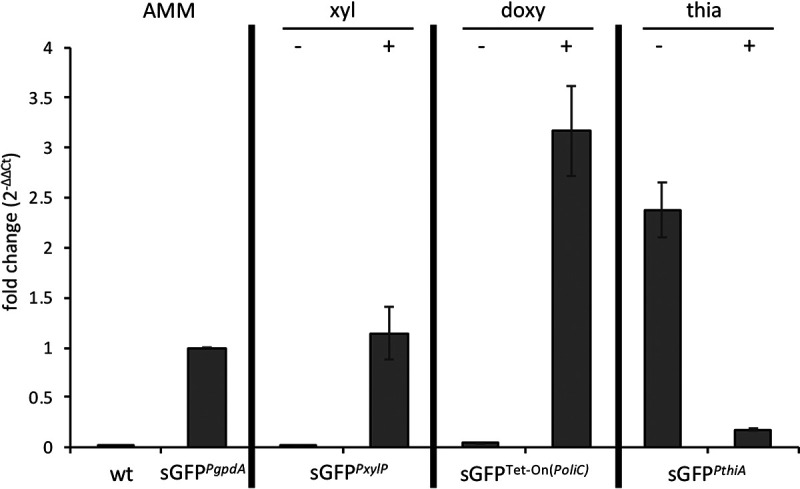
Quantitative real-time PCR assessment of promoters’ tightness and inducibility. Transcriptional evaluation of *PxylP*-, Tet-On(*PoliC*)-, and *PthiA*-driven *sgfp* expression was performed under the respective repressing and inducing conditions. *PxylP* was repressed in AMM in the presence of glucose and induced by supplementing the medium with 1% xylose (xyl). Tet-On(*PoliC*) was repressed in the absence of doxycycline (doxy) and induced by supplementation with 10 μg/mL doxycycline. *PthiA* was induced in AMM and repressed by supplementation with 10 μM thiamine (thia). *sgfp* expression levels were normalized to that generated by *PgpdA*. wt, lacking *sgfp*, served as a negative control.

To precisely determine the promoter leakiness, *sgfp* mRNA levels generated by each promoter system under inducing and noninducing conditions were measured by quantitative real-time PCR (Fig. 2). The wt was included as a negative control, and sGFP*^PgpdA^* was used for normalization. In the absence of xylose, no significant *sgfp* expression could be detected for *PxylP*. For both Tet-On(*PoliC*) without doxycycline and *PthiA* with thiamine *sgfp*, mRNA levels were low but significantly higher than that generated by *PxylP* (*P* < 0.05). During induction, all three promoters showed an expected strong expression. Under control of *PxylP*, *sgfp* transcript levels were comparable to that of *PgpdA* (*P* > 0.05), and *PthiA* as well as Tet-On(*PoliC*)-mediated expression was >2-fold higher than that for this constitutive, high-expressing promoter (*P* < 0.05).

### Evaluation of potential interference between inducing and repressing substances.

**FIG 3:**
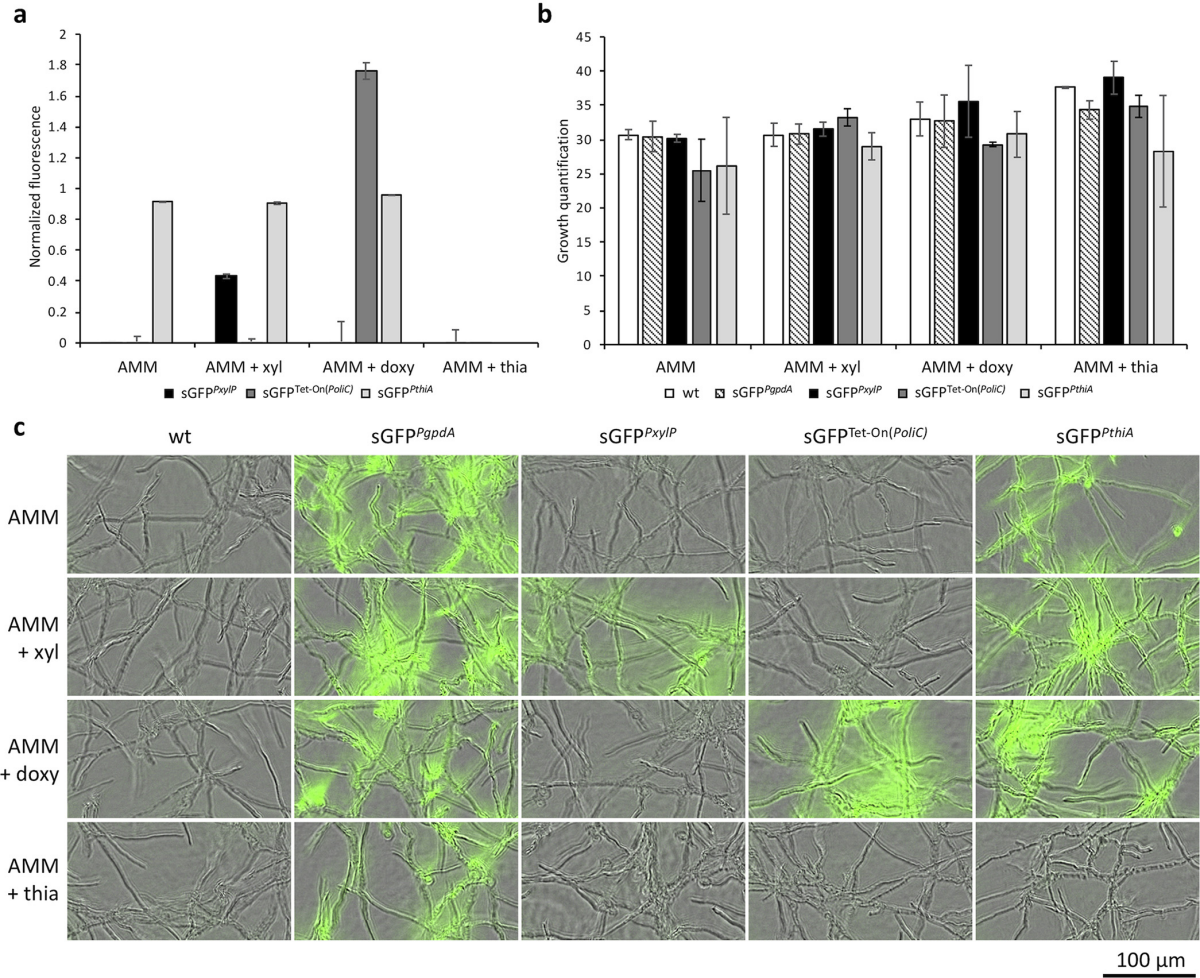
Effects of each specific inducing or repressing compound on the tunability of the other selected promoter systems and on fungal growth. (a) Fluorescence-based assay monitoring sGFP*^PxylP^*, sGFP^Tet-On(^*^PoliC^*^)^, and sGFP*^PthiA^* in the presence of 1% xylose (xyl), 10 μg/mL doxycycline (doxy), or 10 μM thiamine (thia) to exclude any possible interference between the inducible systems. sGFP*^PgpdA^* was used to normalize and compare the fluorescence between strains. To set a baseline and evaluate unspecific GFP fluorescence, the background signal generated by wt was subtracted. (b and c) Confluence measurements (b) and representative images (c) showing fungal growth of the reporter strains in the presence of xyl, doxy, or thia. wt was used as a growth reference and to evaluate unspecific GFP fluorescence generated by hyphae.

Having identified three inducible systems for regulatable gene expression, we sought to further explore the opportunity to utilize them simultaneously, yet still independently from each other. To ensure that gene expression driven by each promoter could be individually tuned, we needed to verify that each condition chosen for either repression or induction of one specific inducible promoter would not interfere with the regulation of the other two. Based on the requirements of each promoter for conditional expression, we selected as a common medium, AMM containing 1% glucose plus the respective inducers (1% xylose or 10 μg/mL doxycycline) or repressor (10 μM thiamine). In this setup, adequate growth of the strains was ensured, *PthiA* drives overexpression of sGFP and *PxylP* displays almost no leakiness. The addition of 1% xylose or 10 μg/mL doxycycline specifically mediated *PxylP*- or Tet-On(*PoliC*)-based overexpression, respectively, while the expression governed by *PthiA* was unaffected (Fig. 3a). In the presence of 10 μM thiamine, activity of none of the promoters led to detectable sGFP production. Our results indicated that the three promoters could be induced or repressed independently, and accordingly, we postulated that all three can be repressed in AMM in the presence of thiamine and induced by supplementing with xylose and doxycycline.

Another important aspect to verify these results concerned the possible effects that inducers or repressors could have on fungal growth, because for fluorescence measurements to be fully comparable we needed to ensure that there were neither delays in the development nor growth defects. For this, plate reader-based optical density measurements as well as fluorescence measurements were performed. As these readings can lead to inaccurate growth quantification in filamentous fungi, we further performed microscopy-assisted confluence and fluorescence analyses to confirm plate reader-based measurements. In this way, we were able to validate the measured growth and visualize sGFP fluorescence (Fig. 3b and c). All strains showed consistent growth at a level comparable to the wt under all tested conditions. Hyphal formation and branching presented no anomalies.

### Modular induction of three different genes.

At this point, we validated the suitability of these three promoter systems in individual strains. To ultimately ensure their applicability for simultaneous, still independent use, we tested their capacity to conditionally express three target genes in a single strain. For this approach, we selected three genes involved in ergosterol biosynthesis, *erg1*, *hmg1*, and *cyp51A*, the overexpression of which are anticipated to confer resistance to specific commercially available antifungal drugs. In order to generate a triple mutant strain carrying an extra copy of each of the selected genes under the control of one of the tested inducible systems, we exploited the three endogenous counterselectable markers, the *fcyB*, *fcyA*, and *uprt* loci, for targeted integration of the respective expression constructs. *erg1* was set under the control of *PthiA* and integrated into the *fcyB* locus, *cyp51A* was cloned downstream of *PxylP* and integrated into the *fcyA* locus, and *hmg1* was placed under regulation of Tet-On(*PoliC*) and integrated into the *uprt* locus (see Fig. S1f in the supplemental material).

The triple mutant strain was then subjected to antifungal susceptibility testing (Table 1). As a representative for the class of azoles, we picked voriconazole; for the class of statins, we used simvastatin; and for the allylamine we used terbinafine. The wt, which was included as a reference, showed MICs after 48 h of 0.5 μg/mL for voriconazole, 25 μg/mL for simvastatin, and 3.13 μg/mL for terbinafine. The MIC values remained unaffected by addition of thiamine, xylose, or doxycycline. Under repressing conditions for all three promoters investigated, i.e., minimal medium containing thiamine, the triple mutant strain displayed identical MICs as the wt for every antifungal. Supplementation of xylose, still in the presence of thiamine, triggered overexpression of *cyp51A* and specifically increased resistance to voriconazole 4-fold. Similarly, the addition of doxycycline, while maintaining thiamine in the medium, resulted in overexpression of *hmg1*, which permitted growth of this strain in the highest simvastatin concentration tested (>100 μg/mL). Overexpression of this gene caused an 8-fold further increase in voriconazole resistance. The withdrawal of thiamine from the medium, instead, led to overexpression of *erg1* as well as specific resistance to all tested concentrations of terbinafine (>100 μg/mL).

**TABLE 1:** Antifungal susceptibility testing comparing wt with the inducible strain *erg1^PthiA^ cyp51A^PxylP^ hmg1*^Tet-On^^(*PoliC*^^)^^*a*^

Drug	Induction	MIC for wt		MIC for *erg1^PthiA^ cyp51A^PxylP^ hmg1^Tet-On^*^(^*^PoliC^*^)^	
		24 h	48 h	24 h	48 h
Voriconazole					
AMM + thia		0.5	0.5	0.5	0.5
AMM + xyl + thia	*cyp51A*	0.5	0.5	2	2
AMM + doxy + thia	*hmg1*	0.5	0.5	4	4
AMM	*erg1*	0.5	0.5	0.5	0.5
Simvastatin					
AMM + thia		12.5	25	12.5	25–50
AMM + xyl + thia	*cyp51A*	12.5	25	12.5	25–50
AMM + doxy + thia	*hmg1*	12.5	25	>100	>100
AMM	*erg1*	12.5	25	12.5	25–50
Terbinafine					
AMM + thia		1.56	3.13	1.56	3.13
AMM + xyl + thia	*cyp51A*	1.56	3.13	1.56	3.13
AMM + doxy + thia	*hmg1*	1.56	3.13	1.56	3.13
AMM	*erg1*	1.56	3.13	>100	>100

a For both strains, voriconazole, simvastatin, and terbinafine MIC values were determined after 24 and 48 h. Specific modular regulation of one of the ergosterol genes was achieved by supplementing AMM with thiamine (thia), xylose (xyl), or doxycycline (doxy).

## DISCUSSION

To date, filamentous fungi play a central role in industrial microbiology as producers of a variety of metabolites and enzymes, including also small bioactive compounds and antibiotics (2, 4, 5, 7, 9, 48, 49). In parallel, diseases revealed to be caused by filamentous fungal pathogens have increased consistently in recent years, and the real numbers are most likely severely underestimated due to undetected or misdiagnosed cases (1, 3, 50, 51). Due to a plethora of advantages, e.g., precise genome editing, efficacy, and targeted DNA delivery, recent decades witnessed an increasing number of applications in fungi. Most of these applications are heavily based on genetic manipulation and synthetic biology, which highlights the critical demand for new and improved tools for assembling and optimizing the genetic design (9). When considering the slightly more complex perspective of a coexpression system, or the possibility to fine-tune gene expression only to a defined degree, the role of regulatable promoters becomes pivotal. Despite the historical background, which has entailed inducible promoters primarily employed in gene overexpression or essential gene characterization, the present trend points to their importance in synthetic biology, to maintain a desired balance between cumulative gene expression, expression ratios, and regulation (48, 52).

In this study, we expanded the genetic toolbox of A. fumigatus with two innovative approaches. First, we characterized in more detail inducible promoter systems available in this species, defining the preferred options and conditions according to the experimental design. Three selected promoters were tested in the same strain for their modular use, revealing the possibility of conditional multigene expression with no interference between the modules. Second, for this approach we exploited and implemented the previously described endogenous counterselectable marker system based on pyrimidine salvage pathway genes (37). We created three plasmids, containing the upstream and downstream regions of markers *fcyB*, *fcyA*, and *uprt* together with the tunable expression cassettes. This allowed, upon linearization, their targeted genomic insertions.

The choice of the selected promoter was driven by the aim to find systems with strong and tunable expression properties, functional in a broad range of filamentous fungi, either used in biotechnological environment or in relation to emerging diseases. A variety of promoters regulated by xylose have been well established in several species, including diverse species of Aspergillus and *Penicillium*, Acremonium chrysogenum, and Trichoderma reesei (27, 53–58). In A. fumigatus, the xylanase promoter *PxylP* from P. chrysogenum has been well characterized and broadly used (27, 38, 42). The tetracycline-regulatable expression systems, after being identified in bacteria, have been modified and optimized for use in eukaryotic organisms, again having major applications among Aspergillus species (19, 20, 28, 59, 60). However, this system also has been successfully used in other industrial and clinically relevant species, such as Fusarium fujikuroi (61), Saccharomyces cerevisiae (62), Schizosaccharomyces pombe (63), and Candida albicans (64). For A. fumigatus, several constructs have been made using tetracycline expression system modules, generating variants that can be repressed (Tet-Off) and variants that can be induced (Tet-On) by tetracycline or doxycycline (19, 20, 45, 46, 65). Considering the number of available versions, we tested in our preliminary analysis two promoter systems, Tet-On(*PoliC*) (45) and Tet-On(*PpkiA*) (46). Based on the objective of our study, i.e., to elucidate promoters suitable for overexpression rather than silencing, we opted for the Tet-On(*PoliC*) version. As a third inducible system, we selected the *thiA* promoter from A. oryzae (29). Thiamine, often referred to as vitamin B_1_, in its pyrophosphate form is considered a universal cofactor and essential to most living organism. It is synthesized in bacteria, fungi, and plants and has to be supplied with the diet in mammals (66). In a previous study, we investigated a point-mutated version of *PthiA* that render the promoter activity independent of thiamine regulation (38). Expression patterns suggested that, when active, *PthiA* should mediate high expression levels. Despite the fact that this promoter has been barely exploited, and for A. fumigatus strictly in relation to virulence and antifungal resistance-related studies (31, 67), we anticipated that its full potential has not yet been discovered.

The three promoter systems were first evaluated separately, to establish the optimal conditions for their induction and repression. *PxylP* was always partially repressed in the presence of glucose but still reached a high level of overexpression in the classical, xylose-containing AMM. Withdrawal of glucose from the medium or substitution with fructose further increased expression >5-fold upon induction. The titration experiment additionally demonstrated excellent tunability of *PxylP*-mediated gene expression. Based on these findings, we decided to use the conventional conditions of 1% glucose for repression and 1% glucose plus 1% xylose for induction. If higher expression were required, however, the option of removing glucose from the medium would be generally available. A similar pattern was identified by analyzing the Tet-On systems. Comparing Tet-On(*PpkiA*) and Tet-On(*PoliC*), both systems appeared suitable for tunable regulation. Nevertheless, by far stronger expression was achieved by the latter. Even in the presence of a substantial amount of inducer, Tet-On(*PpkiA*) was unable to drive high expression. In addition to its excellent overexpression properties, Tet-On(*PoliC*) showed only marginal leakiness under repressing conditions, making this system the favored candidate for a conditional multigene overexpression system. On the other hand, Tet-On(*PpkiA*) could be employed if very low expression is required, for example, in a metabolic step that produces a toxic compound. The thiamine-responsive promoter presented the opposite profile in our titration curve, as increasing concentrations of thiamine proportionally repressed the promoter activity. When fully active, *PthiA* achieved an expression level comparable to the maximal induction of Tet-On(*PoliC*), confirming the hypothesis of being an extremely strong promoter. The expression profile was clearly dose dependent, and a thiamine concentration of only 30 nM was sufficient to completely inhibit *PthiA* in a microtiter plate assay.

As our first application of the newly developed toolbox, we engineered specific steps of the ergosterol biosynthetic pathway by generating the triple mutant *erg1^PthiA^cyp51A^PxylP^hmg1*^Tet-On(^*^PoliC^*^)^. In this strain, we manipulated the expression and therefore enzymatic activity of the three enzymes, squalene epoxidase, sterol 14α-demethylase, and 3-hydroxy-3-methylglutaryl-coenzyme A (HMG-CoA) reductase, the latter catalyzing the initial committed step of ergosterol biosynthesis. Overexpression of each gene specifically increased resistance to the corresponding antifungal agent, which demonstrated the complete independence of each promoter’s inducibility. Overexpression of *hmg1* also increased resistance to voriconazole. As major clinical mechanisms of azole resistance are linked to mutations that are believed to increase Hmg1 activity, this outcome was not surprising (68). In this regard, it has to be mentioned that mutations within the sterol-sensing domain of Hmg1 have been reported to confer azole resistance, most likely due to defective negative regulation of the enzyme (68). In this proof-of-principle experiment, we demonstrated the role of each gene in resistance; however, the described tool is anticipated to allow the tuning of a broad range of biosynthetic pathways.

Taken together, we assessed and combined previously described inducible promoters to establish a conditional multigene expression system that could be utilized to independently express and regulate three different genes in a single strain. Our study provides the necessary data to selectively modify each building block, tailoring this modular tool to achieve the desired cellular performance. As previously demonstrated (37), the pyrimidine salvage pathway appears well conserved evolutionarily, and for targeted integration of expression cassettes, the encoding counterselectable marker loci have proved to be valuable instruments. Altogether, our data suggest a broad applicability of the multigene expression system described here. In addition, various current studies are emphasizing that promoters and other genomic elements are likely to be interchangeable among various filamentous fungi, which indicates the potential heterologous use of our modular inducible multigene expression system in other species (9, 69, 70).

## MATERIALS AND METHODS

### Strains and plasmids.

A. fumigatus mutants produced in this study are derived from the parental strain A1160P+ (71), here referred to as wt. A complete list of all used strains is available in Table S1 in the supplemental material. Gene cassettes for homologous recombination were generated using either a fusion PCR approach or by assembly with NEBuilder (New England Biolabs Inc., Ipswich, MA, USA). Oligonucleotides used in this study are listed in Table S2. The strain sGFP*^PgpdA^*, constitutively expressing sGFP, was used in our experiments as a reference. It was generated by first PCR amplifying the sGFP*^PgpdA^* expression cassette from pFG36 (72) using primers hph-FW and hph-RV. This cassette was then linked with the a 5′- and 3′-*fcyB*-flanking region using fusion PCR as previously described (37) and transformed into the wt. The strain sGFP*^PxylP^* was available in our lab from a previous study (previously known as *fcyB*^GFP^) (37).

The construct to generate sGFP^Tet-On(^*^PoliC^*^)^ was obtained by PCR amplifying the backbone from the template plasmid pfcyB (37) with primers BBdel-FW and BBdel-RV, the Tet-On inducible promoter cassette from the plasmid pJW128 (45) using primers pX-TetON-FW and BBTetON-RV, and the *sgfp* reporter gene from the template pFG36 (72), with primers sGFP-TetOn-FW and sGFPTAnTrpC-Bbdel-RV (Fig. S1a). The construct for the generation of the strain sGFP^Tet-On(^*^PpkiA^*^)^ was produced in a similar way, simply exchanging the inducible promoter cassette with the one amplified from the plasmid pYZ002 (46), using primers pX-TetON-FW and TetON-tight-sGFP-RV. The two assemblies resulted in the new plasmids pΔfcyB_sGFP^Tet-On(^*^PoliC^*^)^ and pΔfcyB_sGFP^Tet-On(^*^PpkiA^*^)^, respectively, which were then transformed into A. fumigatus wt strain after linearization with PmeI.

The construct to generate the strains carrying the sGFP expression gene under the control of *PptrA* was also assembled by connecting different fragments. First, the promoter and terminator regions of the pyrithiamine resistance cassette were PCR amplified from the plasmid pPTR II (TaKaRa Bio, Shiga, Japan) using primer ptrAp_fcyB_fw together with ptrAp_GFP_RV and ptrAt_GFP_fw together with ptrAt_fcyB_RV, respectively. The coding sequence for sGFP was obtained via PCR from the plasmid pFG36 (72) using primers GFP_cds_fw and GFP_cds_RV and assembled with the other two fragments into the previously linearized pfcyB backbone (37) (Fig. S1b). The resulting plasmid, pΔfcyB_sGFP*^PptrA^*, was used as template for site-directed mutagenesis using primers ptrAp_nat_fw and ptrAp_nat_RV, in order to reverse the point mutation in the 5′-UTR that generated resistance to pyrithiamine (29). This way, the native thiamine-responsive promoter of *thiA* was restored. The resulting plasmid was termed pΔfcyB_sGFP*^PthiA^*. Both pΔfcyB_sGFP*^PptrA^* and pΔfcyB_sGFP*^PthiA^* were linearized with NotI and transformed into wt.

The triple mutant strain *erg1^PthiA^cyp51A^PxylP^hmg1*^Tet-On(^*^PoliC^*^)^ was generated stepwise. First, the *erg1* sequence was amplified from wt genomic DNA using primers PptrAerg1-FW and PptrAerg1-RV, and the PCR product was assembled into a backbone obtained by linearizing pΔfcyB_sGFP*^PthiA^* with primers PptrA-BB-FW and PptrA-BB-RV (Fig. S1c). The resulting plasmid, pΔfcyB_erg1*^PthiA^*, was used to transform wt, leading to the generation of the strain *erg1^PthiA^*, which was then used as background to transform the following construct. The plasmid backbones for the next steps, pΔfcyA and pΔuprt, were obtained in a similar way to that previously described for pfcyB (37). Briefly, a pUC19 plasmid was linearized with primers pUC19L-FW and pUC19L-RV and fused with approximately 1 kb of the 5′- and 3′-UTRs of *fcyA* or *uprt*. All flanking regions were amplified from wt genomic DNA. For *fcyA*, the upstream flank was amplified using primers 5′fcyA-FW and 5′fcyA-RV, the downstream region was amplified using primers 3′fcyA-FW and 3′fcyA-RV. Similarly, for *uprt* the upstream and downstream regions were obtained using primers 5′uprt-FW together with 5′uprt-RV and 3′uprt-FW together with 3′uprt-RV, respectively. The primers used to amplify the flanking regions of *fcyA* and *uprt* contained overlapping regions necessary for the assembly with NEBuilder (New England Biolabs Inc., Ipswich, MA, USA) as well as NotI/PmeI restriction sites to linearize the constructs before transformation. The genomic sequences for *cyp51A* and *hmg1* were amplified from wt DNA using primers cyp51Axyl-FW together with cyp51Axyl-RV and hmg1TetON-FW together with hmg1TetON-RV, respectively. *PxylP* and the *trpC* terminator were amplified from the pΔfcyB-PxylP-hph plasmid (38) using primers pX-cass-FW together with pX-RV.2 and pX-FW.2 together with pX-cass-RV, respectively, and Tet-On(*PoliC*) was amplified from pΔfcyB_sGFP^Tet-On(^*^PoliC^*^)^ using primers px-TetON-FW and px-TetON-RV. The fragments encoding *PxylP*, *cyp51A*, and the *trpC* terminator were assembled into the pΔfcyA backbone, which was linearized before with primers BBdel-FW and BBdel-RV (Fig. S1d). The newly generated plasmid pΔfcyA_cyp51A*^PxylP^* was linearized with restriction digestion using NotI and transformed into Δ*fcyB*-*erg1^PthiA^*, thus producing the double mutant strain *erg1^PthiA^cyp51A^PxylP^*. Similarly, the fragments Tet-On(*PoliC*), *hmg1*, and the *trpC* terminator were assembled into the backbone pΔuprt, which was previously linearized with primers BBdel-FW and BBdel-RV (Fig. S1e). The newly generated plasmid, pΔuprt_hmg1^Tet-On(^*^PoliC^*^)^, was linearized with restriction digestion using PmeI and transformed into *erg1^PthiA^cyp51A^PxylP^*, thus producing the triple mutant strain *erg1^PthiA^cyp51A^PxylP^hmg1* ^Tet-On(^*^PoliC^*^)^.

Transformation of A. fumigatus was achieved following the protocol previously described exploiting the counterselectable markers *fcyB*, *fcyA*, and *uprt* (37, 38), and correct integration was validated with Southern blot analysis.

### Nucleic acid manipulation.

Circular plasmids from Escherichia coli were extracted using the Monarch Plasmid Miniprep kit (New England Biolabs Inc., Ipswich, MA, USA). For PCR amplification, Q5 high-fidelity DNA polymerase (New England Biolabs Inc., Ipswich, MA, USA) was employed according to the manufacturer’s instructions.

### Microtiter plate-based reporter assay.

GFP-reporter assays were performed in triplicate using a Greiner Bio-One 96-well polystyrene black microplate with flat bottom and chimney well (Greiner Bio-One International GmbH, Frickenhausen, Germany). Each well contained 100 μL comprising 50 μL of 2× AMM (73) and 50 μL of a 2 × 10^5^/mL spore solution. The concentrated medium was supplemented with glucose, fructose, xylose, thiamine, or doxycycline in order to achieve the desired final concentrations. For the titration assays, the concentration gradient for each compound was obtained via serial dilutions. Fluorescent measurements (GFP excitation at 470/15 nm; emission at 515/20 nm) were carried out after 24 h of incubation at 37°C using the CLARIOstar Plus microplate reader (BMG Labtech, Ortenberg, Germany).

### Growth comparisons between strains.

Nunc 96 plates (Thermo Scientific Inc., Waltham, MA, USA) were used for testing the growth of the sGFP reporter strains in comparison to wt. Spores were diluted to a final concentration of 1 × 10^5^ spores/mL, and each well contained 100 μL. Plates were incubated at 37°C for 24 h and then scanned with the IncuCyte S3 live-cell analysis system equipped with a 20× magnification S3/SX1 G/R optical module (Essen Bioscience Inc., Ann Arbor, MI, USA). Fungal growth was analyzed using the basic analyzer tool of IncuCyte S3 software (version 2020; Essen Bioscience Inc., Ann Arbor, MI, USA) to determine the percent confluence (segmentation adjustment, 0; adjust size, 0). Images in the green fluorescence channel were exported and analyzed with the instrument software to reduce background noise.

### Quantitative real-time PCR.

A. fumigatus strains were grown in 100-mL liquid cultures for 16 h at 37°C. The medium used was AMM (73) with the respective inducing or repressing components. For induction of *PxylP* and Tet-On(*PoliC*), 1% xylose or 10 μg/mL doxycycline was added, respectively. To repress *PthiA*, 10 μM thiamine was added to the medium. RNA extraction was performed with TRI reagent (Merck, KGaA, Darmstadt, Germany) according to the manufacturer’s instructions, and 10 μg of RNA was used for electrophoresis on a 0.6 M formaldehyde agarose gel. The purity of extracted RNA was evaluated in 1% weight/volume (w/v) agarose gels. To prevent DNA contamination, RNA samples that were used as templates for PCRs underwent treatment with DNase I (New England Biolabs Inc., Ipswich, MA, USA) according to the manufacturer’s instructions, followed by a phenol-chloroform extraction. cDNA was generated by GoScript reverse transcriptase (Promega, Madison, Wi, USA) and subjected to real-time PCR using the Luna universal qPCR master mix (New England Biolabs Inc., Ipswich, MA, USA) with 40 ng/well cDNA as template in a QuantStudio 3 real-time PCR system (Applied Biosystems-Thermo Scientific Inc., Waltham, MA, USA). The actin gene of A. fumigatus was chosen as the housekeeper. To calculate the relative fold change of each gene, the 2^−ΔΔ^*^CT^* method was used. Data were normalized to the sGFP*^PgpdA^* strain. Statistical significance was calculated by using Student's *t* test. Primers used for quantitative real-time PCR are listed in Table S2.

### Antifungal activity assay.

Antifungal susceptibility for simvastatin, voriconazole, and terbinafine was tested according to the European Committee on Antimicrobial Susceptibility Testing broth microdilution reference method (74). Due to the presence of thiamine in RPMI medium, which would affect *PthiA*-driven expression, the assays were carried out in AMM. *PxylP* and Tet-On(*PoliC*) were induced using 1% xylose and 1 μg/mL of doxycycline, respectively. *PthiA* was induced by omitting thiamine. For its repression, 10 μM thiamine was added to the medium. MIC values were obtained by visual determination after 24 and 48 h. The wt served as reference for antifungal susceptibility.
