# Supplementary material for: Modular Inducible Multigene Expression System for Filamentous Fungi
Source: Microbiol Spectr. 2022 Nov 9;10(6):e03670-22. doi: 10.1128/spectrum.03670-22 (PMC9769661; doi:10.1128/spectrum.03670-22)
Supplement: Supplemental file 1 — Figure S1, Table S1, and Table S2. Download spectrum.03670-22-s0001.pdf, PDF file, 0.3 MB [file spectrum.03670-22-s0001.pdf]

Supplemental Information

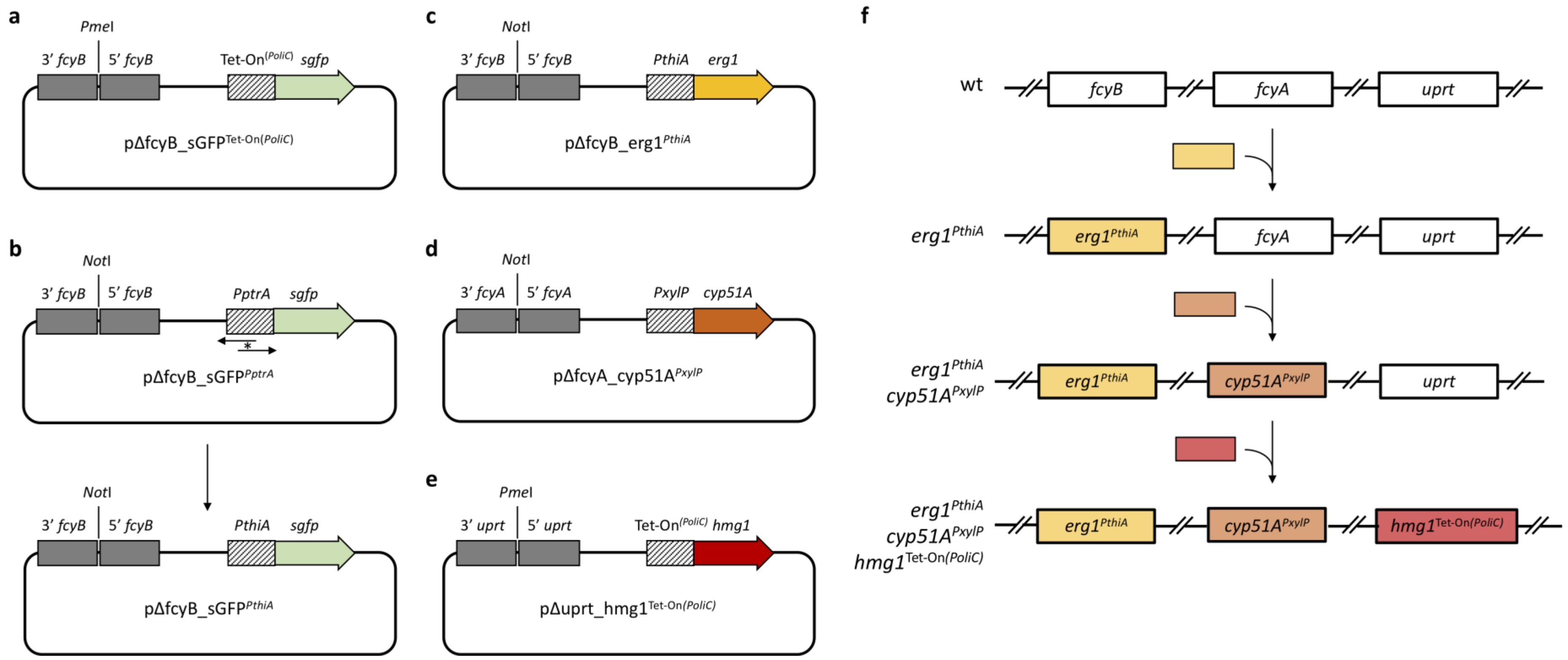

Fig. S1 Schematic representation of the main plasmids and strains generated in this work. (**a**) The plasmid pΔfcyB_sGFP$^{\text{Tet-On}(\textit{PoliC})}$ was obtained by assembling the linearized plasmid pfcyB [1], the Tet-On(*PoliC*) promoter system from the plasmid pJW128 [2] and the sGFP from pFG36 [3]. Upon linearization with *Pme*I it was used to transform *A. fumigatus* wt to generate strain sGFP$^{\text{Tet-On}(\textit{PoliC})}$. (**b**) The plasmid pΔfcyB_sGFP$^{\textit{PptrA}}$ was obtained by assembling the linearized plasmid pfcyB [1], the *PptrA* promoter and terminator from the plasmid ptrII and the sGFP from pFG36 [3]. Upon linearization with *Not*I it was used to transform *A. fumigatus* wt yielding strain sGFP$^{\textit{PptrA}}$. pΔfcyB_sGFP$^{\textit{PptrA}}$ was also used as template to perform site-directed mutagenesis in the 5' UTR of *PptrA* (*) to generate the plasmid pΔfcyB_sGFP$^{\textit{PthiA}}$ carrying the native version of the *thiA* promoter. Upon linearization with *Not*I this plasmid was used to transform *A. fumigatus* wt to produce sGFP$^{\textit{PthiA}}$. (**c**) The plasmid pΔfcyB_erg1$^{\textit{PthiA}}$ was

obtained by replacing the sGFP in pΔfcyB_sGFP^{PthiA} with *erg1* coding sequence, amplified from wt genomic DNA. Upon linearization with *Not*I it was used to transform *A. fumigatus* wt to generate *erg1*^{PthiA}. (**d**) The plasmid pΔfcyA_cyp51A^{PxylP} was obtained by assembling the linearized plasmid pΔfcyA, *PxylP* derived from the plasmid pΔfcyB-PxylP-hph [4] and the *cyp51A* coding sequence amplified from wt genomic DNA. Upon linearization with *Not*I it was used to transform *erg1*^{PthiA} yielding strain *erg1*^{PthiA}*cyp51A*^{PxylP}. (**e**) The plasmid pΔuprt_hmg1$^{\text{Tet-On}(PoliC)}$ was obtained by assembling the linearized plasmid pΔuprt, the Tet-On(*PoliC*) promoter system derived from the plasmid pΔfcyB_sGFP$^{\text{Tet-On}(PoliC)}$ and *hmg1* coding sequence amplified from wt genomic DNA. Upon linearization with *Pme*I it was used to transform *erg1*^{PthiA}*cyp51A*^{PxylP} to produce *erg1*^{PthiA}*cyp51A*^{PxylP}*hmg1*$^{\text{Tet-On}(PoliC)}$. (**f**) Systematic strategy to generate the triple mutant *erg1*^{PthiA}*cyp51A*^{PxylP}*hmg1*$^{\text{Tet-On}(PoliC)}$.

Table S1 Fungal strains used in this study. List of *A. fumigatus* strains used in this study, with the respective genotype and origin.

Strains	Genotype	Reference
wt (A1160P^{+})	Δ*ku80*, *pyrG+*	Fraczek *et al.* (2013) [5]
sGFP^{PgpdA}	Δ*fcyB::PgpdA-sgfp-TtrpC*	this study
sGFP^{PxylP}	Δ*fcyB::PxylP-sgfp-TtrpC*	Birstonas et *al*., (2020) [1]
sGFP$^{\text{Tet-On}(PoliC)}$	Δ*fcyB::*Tet-On(*PoliC*)*-gfp-TtrpC*	this study
sGFP$^{\text{Tet-On}(PpkiA)}$	Δ*fcyB::*Tet-On(*PpkiA*)*-gfp-TtrpC*	this study
sGFP^{PptrA}	Δ*fcyB::PptrA-gfp-TthiA*	this study
sGFP^{PthiA}	Δ*fcyB::PthiA-gfp-TthiA*	this study
ergA^{PthiA}	Δ*fcyB::PthiA-erg1-TthiA*	this study
erg1^{PthiA}*cyp51A*^{PxylP}	Δ*fcyB::PthiA-erg1-TthiA*, Δ*fcyA::PxylP-cyp51A-TtrpC*	this study
erg1^{PthiA}*cyp51A*^{PxylP}*hmg1*$^{\text{Tet-On}(PoliC)}$	Δ*fcyB::PthiA-erg1-TthiA*, Δ*fcyA::PxylP-cyp51A-TtrpC,* Δ*uprT::*Tet-On*(PoliC)-hmg1-TtrpC*	this study

Table S2 List of oligonucleotides used in this study. Primers used in this study to generate plasmids and mutant constructs, as well as for quantitative real-time PCR, listed in order of appearance, and their respective sequence.

Name	Sequence 5'-3'
hph-FW	CCGGCTCGGTAACAGAACTAACGGCGTAACCAAAAGTCAC
hph-RV	GGGAGCATATCGTTCAGAGCTCTTGACGACCGTTGATCTG
Bbdel-FW	TGTGAAATTGTTATCCGCTCACAA
Bbdel-RV	AAACAGCTATGACCATGATTACGC
pX-TetON-FW	AATCATGGTCATAGCTGTTTCCCGTAATCAATTGGCTTCGA
BBTetON-RV	GTGTGATGTGATGGAGTTGAGATG
sGFP-TetON-FW	CCATCTCAACTCCATCACATCACACATGGTGAGCAAGGGCGAGGA
sGFPTAnTrpc-Bbdel-RV	GAGCGGATAACAATTTCACACGCTTACACAGTACACGAGG
TetON-tight-sGFP-RV	TCCTCGCCCTTGCTCACCATGGTGATGTCTGCTCAAGCGG
ptrAp_fcyB_fw	AATCATGGTCATAGCTGTTTGGCAATTGATTACGGGATCCC
ptrAp_GFP_RV	TCCTCGCCCTTGCTCACCATGTTTCAAGTTGCAATGACTATCA
ptrAtGFP_fw	TGGACGAGCTGTACAAGTGAATGACTCACTACCCGAATGGG
ptrAt_fcyB_RV	GAGCGGATAACAATTTCACATGACGATGAGCCGCTCTTGC
GFP_cds_fw	ATGGTGAGCAAGGGCGAGG
GFP_cds_RV	TCACTTGTACAGCTCGTCCATG
ptrAp_nat_fw	GCGAAAAGGATCATGCCTTCTCTCGTTCTTCC
ptrAp_nat_RV	AAGGCATGATCCTTTTCGCTGGTATTATCCAGATCAAGTTTTAGCC
BBgpdA-FW	AGTAGATGCCGACCGCGG
BBgpdA-RV	GGTGATGTCTGCTCAAGCG
PgpdAsGFP-FW	CCGCTTGAGCAGACATCACCATGGTGAGCAAGGGCGAGGA
PgpdAsGFP-RV	TCCCGCGGTCGGCATCTACTTCACTTGTACAGCTCGTCCA
PptrAerg1-FW	TAGTCATTGCAACTTGAAACATGGCTACAACTCCGATCAACG
PptrAerg1-RV	CCATTCGGGTAGTGAGTCATTCAGCAGAAGGCTTCAATTAGC
PptrA-BB-FW	ATGACTCACTACCCGAATGGG
PptrA-BB-RV	GTTTCAAGTTGCAATGACTATCATCTG
pUC19L-FW	GGCATGCAAGCTTGGCGTAA
pUC19L-RV	GTACCGAGCTCGAATTCACTG
5'fcyA-FW	GGTAGCGGCCGCGTTTAAACTTGAAACTCCGAGGAAGTCG
5'fcyA-RV	TTACGCCAAGCTTGCATGCCTATGTGGATCCAGAGCGTCA
3'fcyA-FW	AGTGAATTCGAGCTCGGTACTTCGACAAAATGCCATTGAA
3'fcyA-RV	TCAAGTTTAAACGCGGCCGCTACCTCCCCGAATACCATGA
5'uprt-FW	AGTAGCGGCCGCGTTTAAACGGAAGGACAGGTACGCCATA
5'uprt-RV	TTACGCCAAGCTTGCATGCCCGGAGCACTCTGAAAATTGG
3'uprt-FW	AGTGAATTCGAGCTCGGTACTCCCATCGTGTAGCGACATA
3'uprt-RV	TTCCGTTTAAACGCGGCCGCTACTACCTTCGCCCTCTGGA
cyp51Axyl-FW	ATCGACTCGAAGAACCAACCATGGTGCCGATGCTATGGC
cyp51Axyl-RV	TGAAATCACTGCTGCCATGGTCACTTGGATGTGTTTTTCGACC
hmg1TetON-FW	ATCACACGGCCTGAGTGGCCATGGCTACCTCTCTGATTACAAGG
hmg1TetON-RV	TGAAATCACTGCTGCCATGGTCACTCCGAGGAGGACATCG
pX-cass-FW	AATCATGGTCATAGCTGTTTCTGATGCGAGCAACAGTATGC
pX-RV.2	GGTTGGTTCTTCGAGTCGATG
pX-FW.2	CCATGGCAGCAGTGATTTCA
pX-cass-RV	GAGCGGATAACAATTTCACATGAGGGTTGAGTACGAGATTGG
qRT_act1_for	CCACGTCACCACTTTCAACTC
qRT_act1_rev	CTGCATACGGTCGGAGATAC
RTsGFP-FW1	TCTTCTTCAAGGACGACGGC
RTsGFP-RV1	AAGTCGATGCCCTTCAGCTC

References

1 Birstonas, L. *et al.* Multiplex Genetic Engineering Exploiting Pyrimidine Salvage Pathway-Based Endogenous Counterselectable Markers. *mBio* **11**, doi:10.1128/mBio.00230-20 (2020).

2 Helmschrott, C., Sasse, A., Samantaray, S., Krappmann, S. & Wagener, J. Upgrading fungal gene expression on demand: improved systems for doxycycline-dependent silencing in Aspergillus fumigatus. *Appl Environ Microbiol* **79**, 1751-1754, doi:10.1128/AEM.03626-12 (2013).

3 Sastré-Velásquez L.E., D. A., Kühbacher A., Baldin C., Alcazar-Fuoli L., Niedrig A., Müller C., Gsaller F. The fungal expel of 5-fluorocytosine derived fluoropyrimidines mitigates its antifungal activity and generates a cytotoxic environment. *bioRxiv*, doi:https://doi.org/10.1101/2022.08.24.504767 (2022).

4 Baldin, C. *et al.* Inducible Selectable Marker Genes to Improve Aspergillus fumigatus Genetic Manipulation. *J Fungi (Basel)* **7**, doi:10.3390/jof7070506 (2021).

5 Fraczek, M. G. *et al.* The cdr1B efflux transporter is associated with non-cyp51a-mediated itraconazole resistance in Aspergillus fumigatus. *J Antimicrob Chemother* **68**, 1486-1496, doi:10.1093/jac/dkt075 (2013).
